# Detection and characterization of *Lactobacillus* spp. in the porcine seminal plasma and their influence on boar semen quality

**DOI:** 10.1371/journal.pone.0202699

**Published:** 2018-09-07

**Authors:** Martin Schulze, Jana Schäfer, Christian Simmet, Markus Jung, Christoph Gabler

**Affiliations:** 1 Institute for the Reproduction of Farm Animals Schönow, Bernau, Germany; 2 Minitüb, Tiefenbach, Germany; 3 Institute of Veterinary Biochemistry, Department of Veterinary Medicine, Freie Universität Berlin, Berlin, Germany; Universite Clermont Auvergne, FRANCE

## Abstract

The presence of pathogenic bacteria in ejaculates has been a topic in boar semen preservation over the last decades. Since little information is available on commensal bacteria in boar semen, the aim of the present study was to identify commensal lactobacilli in fresh cryopreserved boar semen and to examine their influence on boar semen quality. Therefore, 111 boar ejaculates were investigated for the presence of *Lactobacillus* species. Thirty samples (27%) contained viable *Lactobacillus* species (e.g. *L*. *amylovorus*, *L*. *animalis*, *L*. *reuteri and Weisella minor*). *L*. *animalis* and *L*. *buchneri* DSM 32407 (isolated from the bovine uterus) qualified for further examinations based on their growth rate in six antibiotic-free boar semen extenders. After a 120 min short-term incubation with an antibiotic-free BTS-extender, progressive motility was diminished (*P* = 0.001) upon addition of 10^5^ and 10^6^ colony forming units (CFU/mL) *L*. *animalis*. The supplementation with *L*. *buchneri* DSM 32407 had no significant (*P* > 0.05) influence on sperm quality during short-term co-incubation. After 168 h long-term co-incubation, motility analysis revealed a negative (*P* = 0.026) impact of 10^5^ CFU/mL *L*. *buchneri* DSM 32407. A concentration- and storage-dependent effect is particularly obvious (*P* < 0.001) using 10^6^ CFU/mL *L*. *buchneri* DSM 32407. Most notably, the thermo-resistance (TRT) for 10^6^ CFU/mL *L*. *buchneri* DSM 32407 (*P* = 0.001) was inferior to BTS with and without gentamicin after 72 and 168 h of semen co-incubation. The supplementation of 10^5^ CFU/mL *L*. *buchneri* DSM 32407 impaired progressive motility to a lesser extent. The percentage of mitochondrially active spermatozoa after 96 h (*P* = 0.009) and membrane-intact spermatozoa after 168 h (*P* < 0.001) was lower when 10^6^ CFU/mL *L*. *buchneri* DSM 32407 were suspended compared with all other groups. Finally, the addition of *L*. *buchneri* DSM 32407 to BTS-extended boar semen had no competitive effect on the total amount of bacteria 48 h after co-incubation. In summary, the present study demonstrated that there are *Lactobacillus* species present in the porcine seminal plasma, which can be cultivated using standard procedures. However, long-term co-incubation of lactic acid bacteria with spermatozoa had a negative influence on spermatozoa.

## Introduction

In the swine industry, artificial insemination (AI) is a common reproductive technology to apply spermatozoa to the female reproductive tract. Therefore, there is a great need to use pathogen-free semen. Due to the natural bacterial content of fresh boar semen, measures to control bacterial growth during storage of insemination doses are necessary. This reduces the risk of potential bacterial contamination of the uterus. Virtually all ejaculates collected from healthy donors contain bacteria stemming from natural occurrence within the male reproductive tract. Bacterial load in raw semen commonly ranges between 10^4^ and 10^6^ CFU/mL [1]. In addition to animal origins, bacteria from the environment may contaminate semen during collection or processing in the laboratory [2]. Consequences of bacterial contamination predominantly reside in loss of sperm motility, and induction of sperm agglutination and membrane damage, resulting in poor fertility and high economic losses in sow herds [3,4].

Antimicrobial agents are considered to be essential to control bacterial growth in extended boar semen, especially if the AI doses are stored at mesophilic temperatures (15 to 18°C) for several days [5]. For decades, penicillin and streptomycin was the common antimicrobial combination for this purpose [6]. Currently, aminoglycosides, especially gentamicin, are most popular [7]. However, some bacteria have acquired resistance to one or more of these antibiotics and reports have revealed bacteriospermia in 14.7% to 31.2% of extended porcine semen samples [1,8]. Mostly, these resistant bacteria are from the families *Enterobacteriaceae*, *Alcaligenaceae*, and *Xanthomonadaceae* [9]; usually attributed to the environment but also occurring as opportunistic pathogens associated with nosocomial infections in human and animal medicine [10]. To avoid the use of antibiotics, more and more alternative strategies to conventional antibiotics for minimizing risk of developing bacterial resistance come into the focus. These strategies include physical removal of bacteria by colloid centrifugation [11], hypothermic semen storage below 16°C [12], automated semen collection [13], microfiltration of seminal plasma [14], use of antimicrobial peptides [15,16] and essential oils [17].

In the last decades, numerous studies were published concerning the presence of bacteria in the semen of boars worldwide [2,8,9]. The major concern is pathogenic bacteria causing infectious diseases, e.g. brucellosis, chlamydophilosis and leptospirosis [18,19]. Furthermore, an increase in the variety of bacterial species was detected, which also decreased the survival of boar spermatozoa during storage. These enteric bacterial species included strains of *Escherichia coli*. It was shown that these bacterial strains influenced the quality of porcine spermatozoa [20]. In addition, some strains from the families *Enterobacteriaceae*, *Alcaligenaceae* and *Xanthomonadaceae* did not change the sperm morphology but negatively affected other parameters, e.g. acrosome integrity, sperm capacitation or osmotic resistance [21–24]. However, some other species had no significant influence during storage, although these bacteria caused a moderate decrease in pH [9].

Among commensal bacteria, which are defined as having no negative influence on the host, are the group of lactic acid bacteria (LAB) comprising the genera *Lactobacillus*, *Lactococcus*, *Pediococcus*, *Streptococcus*, and *Enterococcus*. The genus *Lactobacillus* contains currently over 180 species and encompasses a wide variety of organisms. *Lactobacilli* are Gram-positive, rod-shaped, non-spore-forming bacteria that are present in the female genital tract of several species [25–29]. It was shown that co-culturing of bovine endometrial epithelial cells with *L*. *buchneri* (now registered as *L*. *buchneri* DSM 32407) up to a multiplicity of infection (MOI) of 10 did not affect the viability of epithelial cells [27]. In addition, the mRNA expression or release of pro-inflammatory factors was not influenced for up to 6 h and 48 h, respectively. In contrast, the presence of *L*. *ruminis* and *L*. *amylovorus* provoked a pro-inflammatory response of the epithelial cells. An early study indicated that LAB have an immuno-stimulatory effect on the endometrium [30]. In that study, after the intrauterine administration of two live *Lactobacillus* spp., an infiltration with mostly mononuclear cells into the endometrium was observed and a colonization of the endometrium by the selected lactobacilli strains for up to 12 days was noted. Beneficial effects of the topical administration of live cultures of LAB on intestinal and/or genital health have been shown for several species including pigs [31], humans [32,33], and cows [34]. In pigs, different lactobacilli strains were isolated from sow milk and their probiotic potential was evaluated through different assays, including survival in conditions simulating those existing in the gastrointestinal tract, production of antimicrobial compounds, adherence to intestinal mucin, production of biogenic amines, degradation of mucin, and pattern of antibiotic sensitivity [35].

In contrast to research on the porcine female, LAB have so far not been the focus of studies concerning the porcine male reproductive tract and their influence on sperm quality is unclear. Therefore, the aim of this study was to isolate and characterize LAB from boar ejaculates, to examine their survival in different boar semen extenders, and investigate their influence on sperm parameters in short- and long-term boar semen co-incubation.

## Material and methods

### Chemicals

All chemicals used in this study were of analytical grade. Unless stated otherwise, they were purchased from Merck (Darmstadt, Germany) or Carl Roth (Karlsruhe, Germany).

### Isolation, identification, and cultivation of different LAB in fresh cryopreserved boar semen

The experimental protocol was approved by the Ethics Committee of our institution (Reg. 2016/02). This ethics committee was known as Bioethics Commission of the IFN (Bernau, Germany). Present study is not an animal experiment. Therefore there is no requirement of disclosure or permit according to German law concerning animal shelter. For routine semen production, ejaculates are sampled at scheduled days. At these days we retrieved material for our studies by splitting ejaculates.

A total of 111 ejaculates obtained from eight AI boar studs (14 ± 2 samples each) in Germany were examined in this study. Only fertile Pietrain boars without signs of genital diseases were chosen for sample collection. The average age (mean ± SD) of the boars was 18.5 ± 5.3 months. All boars were routinely used for production of AI doses, received commercial feed (pellets) for AI boars, and were housed in individual pens equipped with nipple drinkers according to the European Commission Directive for Pig Welfare. Protocols were carried out according to general guidelines for semen processing used in AI studs participating in a quality control audit of the Institute of the Reproduction of Farm Animals Schönow (Bernau, Germany) [36].

Ejaculates were collected randomly by the gloved-hand method. The first phase of the ejaculates was discarded and the gel fraction of the semen was removed by gauze filtration. The collection frequency of ejaculates did not extend for three collections within 2 weeks with at least 3 days of rest in between. An aliquot (2 mL) of each ejaculate was frozen with 400 μl glycerol, transported to the laboratory in liquid nitrogen, and stored at -80°C until microbial analyses. Samples were cultured aerobically or anaerobically on Rogosa SL agar and LBS agar (both Sigma-Aldrich, Steinheim, Germany) at 37°C for up to 72 h to detect lactobacilli as described previously [27]. All isolates were stored at -80°C in MRS broth containing 15% (v/v) glycerol.

Bacterial species were first identified on the agar plates by the characteristics of colony morphology and catalase reaction. Only colonies with a negative catalase reaction were used for culturing in MRS broth. For a first screening, a PCR was carried out using a *Lactobacillus*-specific primer pair [37] (for: 5’-AGC AGT AGG GAA TCT TCC A-3’; rev: 5’- ATT C/TCA AAG CTA CAC ATG-3’; synthesized by Eurofins MWG, Ebersberg, Germany) based on the 16S ribosomal ribonucleic acid (16S rRNA) gene as previously described [27]. The forward and reverse sequences of the PCR products were obtained by a commercial sequence service (GATC Biotech, Konstanz, Germany) followed by comparing the resulting sequences with the NCBI database to identify the specific bacterial species.

Isolated lactobacilli strains from boar semen and one lactobacillus strain isolated from the bovine uterus (*L*. *buchneri*; now registered as *L*. *buchneri* DSM 32407) [27] were prepared for co-incubation with porcine spermatozoa. Only isolated LAB, which showed sufficient growth characteristics in MRS broth could be used for further experiments. Bacteria from glycerol stocks were grown in MRS broth at 37°C up to an optical density of one at the wavelength of 600 nm. Bacterial cells were harvested by centrifugation for 10 min at 3,800 × g, washed once with PBS, re-suspended in PBS, and stored in aliquots at -80°C. The number of colony-forming units (CFU)/mL in the aliquots was determined by plate counting on MRS agar after thawing.

Bacteria, grown in sufficient amount for subsequent experiments, were further characterized with PCR using phylogenetic 16S rDNA primers [27,38]. This allowed the identification of the specific bacterial strains by sequencing (GATC Biotech) of extended amplicons. Briefly, the reaction mixture contained 200 ng of genomic bacterial DNA and the following primer pair (synthesized by Eurofins MWG): for: 5’-AGA GTT TGA TCC TGG CTC AG-3’; rev: 5’-AAG GAG GTG ATC CAG CC-3’.

### Incubation of selected LAB in different boar semen extenders

The suitability of different LAB to survive in various extenders was determined. Four selected LAB (*L*. *reuteri*, *L*. *animalis*, *Weissella minor*, and *L*. *buchneri* DSM 32407) were added to 90 ± 1 mL of six antibiotic-free extenders (Beltsville Thawing Solution (BTS), Merck III, Androhep Plus, Androstar Plus, Androstar Premium and Androstar CryoPlus; all Minitüb, Tiefenbach, Germany) in a final concentration of 10^3^ CFU/mL each. All samples were stored in 100 mL plastic bottles (Minitüb) for boar semen at 17°C for 48 h. Subsequently, 100 μL of each sample were plated on MRS agar and incubated at 37°C under aerobic conditions for 48 h to determine concentrations of viable LAB by rating with a valuation code as follows: ≤10^1^ CFU/mL = 0, ≤10^2^ CFU/mL = 1, ≤10^3^ CFU/mL = 2, ≤10^4^ CFU/mL = 3, ≤10^5^ CFU/mL = 4, and >10^5^ CFU/mL = 5. The incubation of these distinct bacterial strains in each semen extender was repeated independently three times.

### Short- and long-term incubation of selected LAB with porcine spermatozoa in antibiotic-free BTS extender

Ejaculates were collected as described above from 24 fertile Pietrain boars housed at a commercial AI center in northern Germany. Twelve randomly selected ejaculates were used for short- (≤ 120 min) and long-term (≤ 7d) incubation with different LAB in a split-sample procedure, respectively. Only ejaculates that passed minimum requirements for commercial use in AI were included. Criteria for selection of ejaculates stipulated a minimum of 75% morphologically normal spermatozoa, total sperm motility of at least 70%, and a total amount of ≥30 x 10^9^ spermatozoa per ejaculate. The day of semen collection is specified as day 0 (d0) of analysis.

Sperm concentration was adjusted to 24 x 10^6^ spermatozoa/mL with an antibiotic-free Beltsville Thawing Solution (BTS) extender (Minitüb, Germany) using a NucleoCounter SP-100 (Chemometec, Denmark). The dilution of full ejaculates (total volumes between 1,500 and 3,400 mL) was completed at one step at 32 ± 1°C. BTS supplemented with 250 μg/mL gentamicin and BTS without gentamicin were used as positive (+) and negative (-) controls, respectively.

For the short-term incubation to a maximum of 120 min, extended semen was subsequently co-cultured with selected LAB from the previous experiments: *L*. *animalis* (10^5^ and 10^6^ CFU/mL) and *L*. *buchneri* DSM 32407 (10^5^, 10^6^ and 10^7^ CFU/mL) for up to 120 min at 38°C in 10 mL tubes, respectively. For the long-term incubation to a maximum of 168 h, extended semen was co-cultured with 10^5^ and 10^6^ CFU/mL *L*. *buchneri* DSM 32407 and filled in 100 mL plastic bottles (Minitüb) for boar semen. Filling volume was 90 ± 1 mL. The final concentration of *L*. *buchneri* DSM 32407 in this approach was chosen according to the results of the short-term incubation. Finally, all extended samples were placed in a temperature-controlled box at 21°C for 90 min and subsequently stored in a temperature-controlled cabinet at 17°C for up to seven days.

### Monitoring of pH

pH was determined using the Microprocessor pH-meter pH 537 (WTW, Weilheim, Germany). After 120 min short-term incubation at 38°C, pH was measured in BTS-extended semen in the presence of selected LAB. In the long-term experiment, measurement of pH was done after 24, 48, and 72 h of semen incubation with selected LAB at 17°C. After 168 h semen storage in the presence of LAB at 17°C, an aliquot of 10 mL was incubated at 38°C for up to 300 min to measure changes of pH during this period.

### Evaluation of sperm motility

To determine the short-term effects, sperm motility was evaluated after 10, 60 and 120 min incubation. For the long-term effects, an aliquot of 1.5 mL was removed after 24, 48, 96 and 168 h of semen storage with selected LAB and incubated for 10 min at 38°C to assess sperm motility. To assess sperm longevity at body temperature, a thermo-resistance test (TRT) was performed after 72 and 168 h of semen incubation with selected LAB. For this test, an aliquot of 10 mL was incubated at 38°C in a water bath (GFL 1002, Burgwedel, Germany) under air access. After 30 and 300 min incubation, sperm motility was determined. In all experiments, sperm motility was evaluated using the CASA system AndroVision (Minitüb) as described previously [39].

### Flow cytometric evaluation of mitochondrial status and membrane integrity

Sperm viability and mitochondrial activity were assessed in the short-term approach after 120 min at 38°C and in the long-term approach after 48 h and 96 h of semen incubation with selected LAB by double-staining with rhodamine 123 (R123)/propidium iodide (PI; both Sigma-Aldrich) and flow cytometry as described previously [40].

A triple-stain flow cytometric method using PI (Sigma-Aldrich), fluorescein-isothiocyanate conjugated peanut agglutinin (FITC-PNA) and *Pisum sativum* agglutinin (FITC-PSA; both Axxora, Lörrach, Germany) was used in the short-term experiment after 120 min at 38°C and in the long-term experiment after 72 h and 168 h of semen incubation with selected LAB. This technique makes it possible to distinguish viable (intact plasma membrane) from dead spermatozoa and to characterize membrane integrity in the acrosomal region as described previously [41].

### Evaluation of total bacterial cell and LAB amount under aerobic conditions

A dilution series of 1:10^2^ to 1:10^6^ for each semen sample with selected LAB was performed after 48 h long-term incubation to determine the total bacterial load. From each dilution, 100 μL were plated on plate count agar and MRS agar (both Oxoid, Wesel, Germany), incubated at 37°C for 48 h under aerobic conditions, and CFU/mL was recorded. The total bacterial load was calculated by subtraction of the CFU/ml of the plate count agar from the CFU/ml of the MRS agar.

### Statistical analysis

One-way analysis of variance (ANOVA) was carried out using IBM SPSS Statistics 25 (SPSS, Chicago, USA, IL). The ANOVA was used to evaluate the effects of different LAB (treatment) on boar semen quality and total bacterial load. When ANOVA revealed a significant treatment effect, the values were compared using the Tukey-test (*post hoc*). All values are presented as mean ± standard error (SEM). Differences among means were considered to be significant, when *P*-values were < 0.05.

## Results

### Identification of LAB species in boar ejaculates

Ejaculates from 81 (73%) mature Pietrain boars out of a total number of 111 collected samples were culture-negative under selective conditions for lactobacilli. Ejaculates from 24 (21.6%) semen samples contained one LAB strain, and from 6 (5.4%) samples more than one LAB strain. This was confirmed by sequencing with *Lactobacillus* specific primers. All culture-positive samples showed slight bacterial content (<10 colonies). The name of the obtained bacterial species as well as their frequency of occurrence is listed in Table 1. Bacteria, which grow in sufficient amount for subsequent experiments, were subjected to a PCR using phylogenetic 16S rDNA primers. The identity of the species with a percentage of homology to a distinct strain of the NCBI database is listed in Table 2. In the following, the obtained bacterial strains will be named according to these homologous sequences.

**Table 1:** Qualitative characterization of isolated lactic acid bacteria in 111 boar ejaculates.

Identified Species	Frequency
*Lactobacillus* spp.	**20x**
*Lactobacillus reuteri*	**8x**
*Lactobacillus amylovorus*	**2x**
*Weisella minor strain*	**2x**
*Lactobacillus animalis*	**1x**
*Lactobacillus curvatus*	**1x**
*Lactobacillus plantarum*	**1x**
*Lactobacillus vaginalis*	**1x**
*Lactococcus lactis*	**1x**
*Weissella paramesenteroides*	**1x**

**Table 2:** Characterization of isolated uterine lactic acid bacteria in boar ejaculates.

Identified Species	Type Strain	% Identity to Type Strain	Accession #
*L*. *animalis*	FR12	**97%**	KU587454.1
*L*. *reuteri*	B-SW1	**99%**	LC369122.1
*Weisella minor*	JCM 1168	**98%**	LC065034.1

### Growth of selected LAB in different boar semen extenders as culture media

No colonies were observed for *Weisella minor* on agar plates after incubation in all chosen boar semen extenders. *L*. *reuteri* grew on MRS agar with the growth rating 4 out of 5 only after incubation in Androstar CryoPlus, but not after incubation in the other used extenders. In contrast, *L*. *animalis* and *L*. *buchneri* DSM 32407 were able to form colonies on the agar plates independent of the used extender. However, the number of resulting colonies differed between extenders. The rating of the obtained colonies for *L*. *animali*s on the MRS agar plates was after incubation in the semen extenders as follows: rating of 2 for Androstar Plus, rating of 3 for Androstar Premium and Androhep Plus, rating of 4 for BTS and Merck III. *L*. *buchneri* DSM 32407 showed a similar number of obtained colonies with a rating of 4 after incubation in the following extenders: Androstar Plus, Androstar Premium, Androhep Plus, BTS, and Merck III. The number of obtained colonies after incubation in Androstar CryoPlus was rated with the highest values of 5 for *L*. *animalis* and *L*. *buchneri* DSM 32407, respectively. Therefore, *L*. *animalis* and *L*. *buchneri* DSM 32407 were used for co-incubation with spermatozoa in antibiotic-free BTS, the industry’s most commonly used short-term extender for boar semen preservation, in further experiments.

### Effect of *L*. *animalis* and *L*. *buchneri* DSM 32407 on boar sperm quality during short-term incubation

The effect of *L*. *animalis* and *L*. *buchneri* DSM 32407 added to BTS-extended boar semen in concentrations of 10^5^ to 10^7^ CFU/mL is shown in Table 3 for short-term incubation up to 120 min at 38°C. The incubation with *L*. *buchneri* DSM 32407 had no significant influence on progressive motility. However, the progressive motility was influenced significantly (*P* = 0.001) in a negative manner in presence of 10^6^ CFU/mL *L*. *animalis* after 60 min and of 10^5^ CFU/mL *L*. *animalis* after 120 min of incubation at 38°C. The percentage of mitochondrially active and membrane-intact spermatozoa was not affected by the presence of either LAB.

**Table 3:** Effect of different concentrations (CFU/mL) of *L*. *animalis* and *L*. *buchneri* DSM 32407 during short-term incubation on progressive motility, pH, mitochondrial and membrane status of extended boar semen.

Parameter	Incubation (min)	BTS without gentamicin (Control)	*L*.* animalis* (10^5^ CFU/mL)	*L*.* animalis* (10^6^ CFU/mL)	*L*.* buchneri* DSM 32407 (10^5^ CFU/mL)	*L*.* buchneri* DSM 32407 (10^6^ CFU/mL)	*L*.* buchneri* DSM 32407 (10^7^ CFU/mL)	*P value*
Progressive motility (%)	10	84.4 ± 4.4^a^	83.5 ± 3.2^a^	82.2 ± 4.5^a^	84.8 ± 2.5^a^	81.8 ± 6.4^a^	82.4 ± 3.8^a^	0.993
	60	82.4 ± 3.2^a^	80.6 ± 5.4^a^	27.2 ± 13.2^b^	82.6 ± 3.2^a^	81.9 ± 3.1^a^	80.4 ± 2.5^a^	0.001
	120	80.1 ± 6.4^a^	70.2 ± 0.4^b^	22.8 ± 9.8^c^	78.0 ± 5.7^a^	78.3 ± 3.5^a^	78.2 ± 4.5^a^	0.001
Mitochondrially active spermatozoa (%)	120	82.9 ± 3.5^a^	83.5 ± 3.7^a^	81.4 ± 3.0^a^	81.8 ± 4.6^a^	81.2 ± 4.6^a^	78.8 ± 8.0^a^	0.984
Membrane-intact spermatozoa (%)	120	74.3 ± 5.2^a^	73.5 ± 5.9^a^	71.7 ± 4.2^a^	74.1 ± 6.1^a^	73.8 ± 5.8^a^	73.3 ± 5.1^a^	0.998
pH-value	120	7.70 ± 0.02^a^	7.57 ± 0.03^b^	7.56 ± 0.04^b^	7.60 ± 0.03^b^	7.60 ± 0.02^b^	7.49 ± 0.02^c^	0.002

Normospermic ejaculates of 12 fertile boars were extended to 2.4 × 10^7^ spermatozoa/mL in BTS (Minitüb, Germany) without gentamicin. Extended semen was subsequently incubated with different lactobacilli strains and concentrations for 10, 60 and 120 min at 38°C in 10 mL tubes. Values are expressed as mean ± SEM (n = 12 per group). Values with different superscripts within a row differ significantly (*P* < 0.05).

Measurement of pH-value revealed a significant (*P* = 0.002) difference between control and treatment groups. In all cases, the incubation with LAB led to an acidification of the samples. The strongest effect was noted when using 10^7^ CFU/mL *L*. *buchneri* DSM 32407. The adverse effect of *L*. *animalis* on motility already in short-term incubation brought about the decision to conduct long-term incubation only with *L*. *buchneri* DSM 32407. Only 10^5^ and 10^6^ CFU/mL were taken into account, because of the moderate acidification when 10^7^ CFU/mL was used.

### Effect of *L*. *buchneri* DSM 32407 on boar sperm quality during long-term incubation

**Fig 1:**
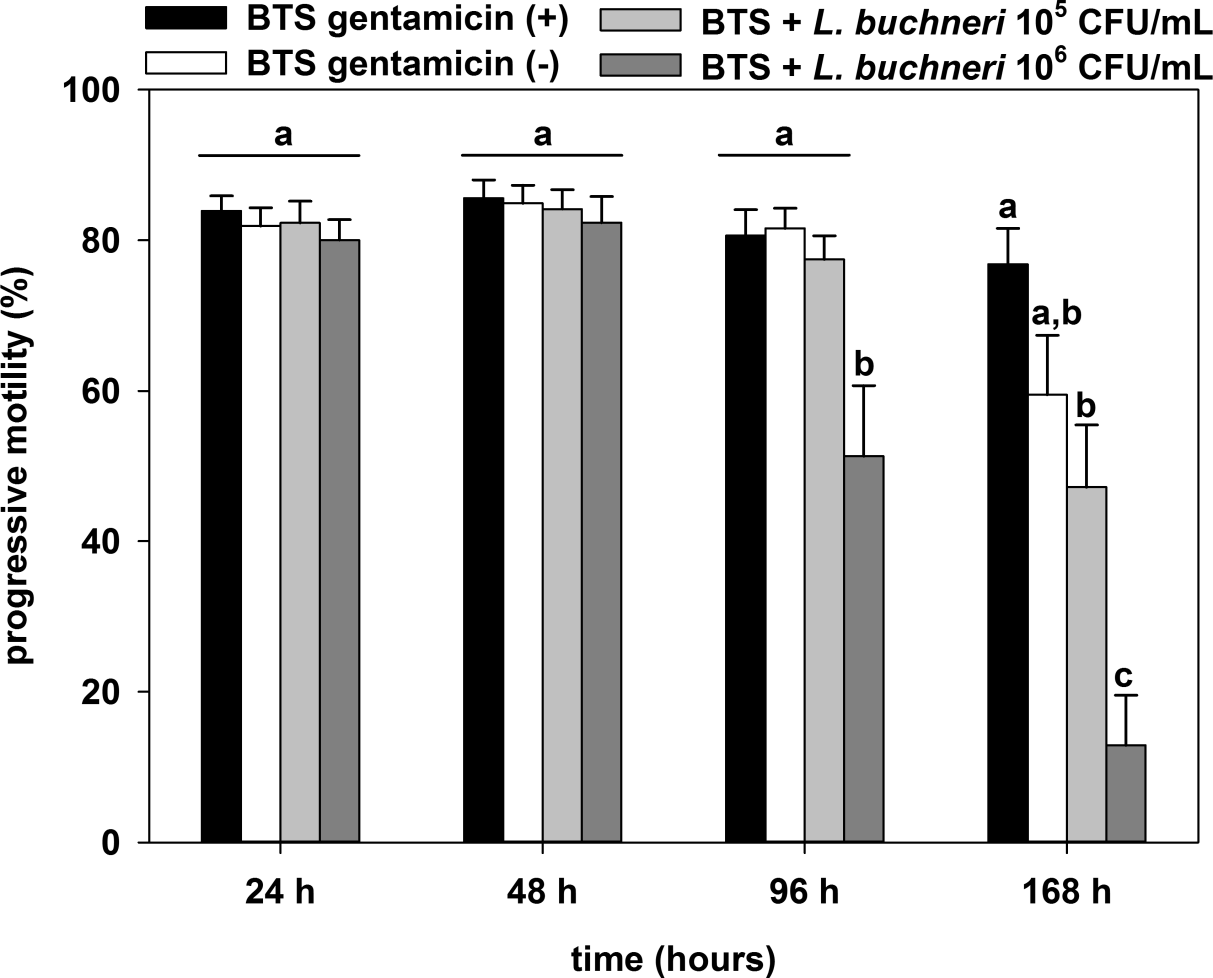
Effect of 10^5^ and 10^6^ CFU/mL *L*. *buchneri* DSM 32407 during long-term incubation in BTS-extended boar semen on progressive motility. Normospermic ejaculates of 12 fertile boars were extended to 2.4 × 10^7^ spermatozoa/mL in BTS with 250 μg/mL gentamicin (positive control (+)) and without gentamicin (negative control (-)). Extended semen was stored in 100 mL semen bottles (Minitüb) for seven days at 17°C. Results are presented as mean ± SEM (n = 12 per group). Bars with different letters indicate significant differences between groups (*P* < 0.05).

There was no difference (*P* > 0.05) in progressive motility between treatment and control groups 24 h and 48 h after incubation (Fig 1). After 96 h of semen incubation with *L*. *buchneri* DSM 32407, a negative impact (*P* = 0.034) of 10^6^ CFU/mL was observed compared with the other groups and this negative effect on progressive motility was even enhanced after 168 h of co-incubation (*P* < 0.001). The motility analysis also revealed a significant (*P* = 0.026) difference between BTS with gentamicin and 10^5^ CFU/mL *L*. *buchneri* DSM 32407 after 168 h of semen incubation. However, no significant difference between BTS w/o gentamicin and 10^5^ CFU/ml *L*. *buchneri* DSM 32407 was observed.

**Fig 2:**
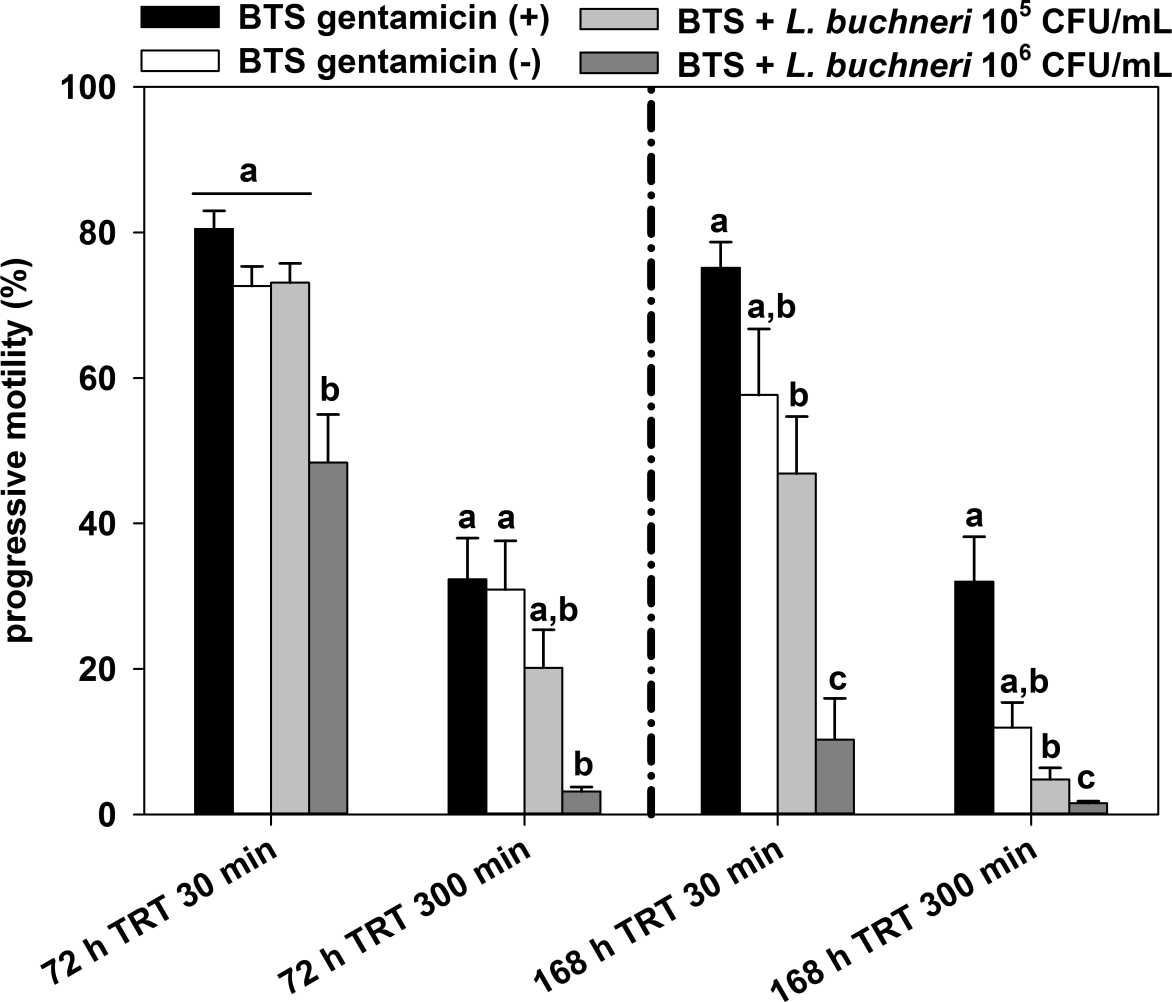
Effect of 10^5^ and 10^6^ CFU/mL *L*. *buchneri* DSM 32407 during long-term incubation in BTS-extended boar semen on progressive motility in a thermo-resistance test (TRT: incubation at 38°C for 30 and 300 min) after 72 and 168 h of semen storage. Normospermic ejaculates of 12 fertile boars were extended to 2.4 × 10^7^ sperm/mL in BTS with 250 μg/mL gentamicin (positive control (+)) and without gentamicin (negative control (-)). Extended semen was stored in 100 mL semen bottles (Minitüb) for seven days at 17°C. Results are represented as mean ± SEM (n = 12 per group). Bars with different letters indicate significant differences between groups (*P* < 0.05).

The TRT yielded similar results (Fig 2). Supplementation of 10^6^ CFU/mL *L*. *buchneri* DSM 32407 resulted in a significantly (*P* = 0.001) lower progressive motility compared with BTS with gentamicin and BTS w/o gentamicin 72 h and 168 h after co-incubation. The concentration of 10^5^ CFU/mL *L*. *buchneri* DSM 32407 impaired progressive motility to a lesser extent, but the negative effect was significant (*P* = 0.024) in TRT after 168 h of semen storage. No significant difference between BTS w/o gentamicin and 10^5^ CFU/ml *L*. *buchneri* DSM 32407 was noted.

**Fig 3:**
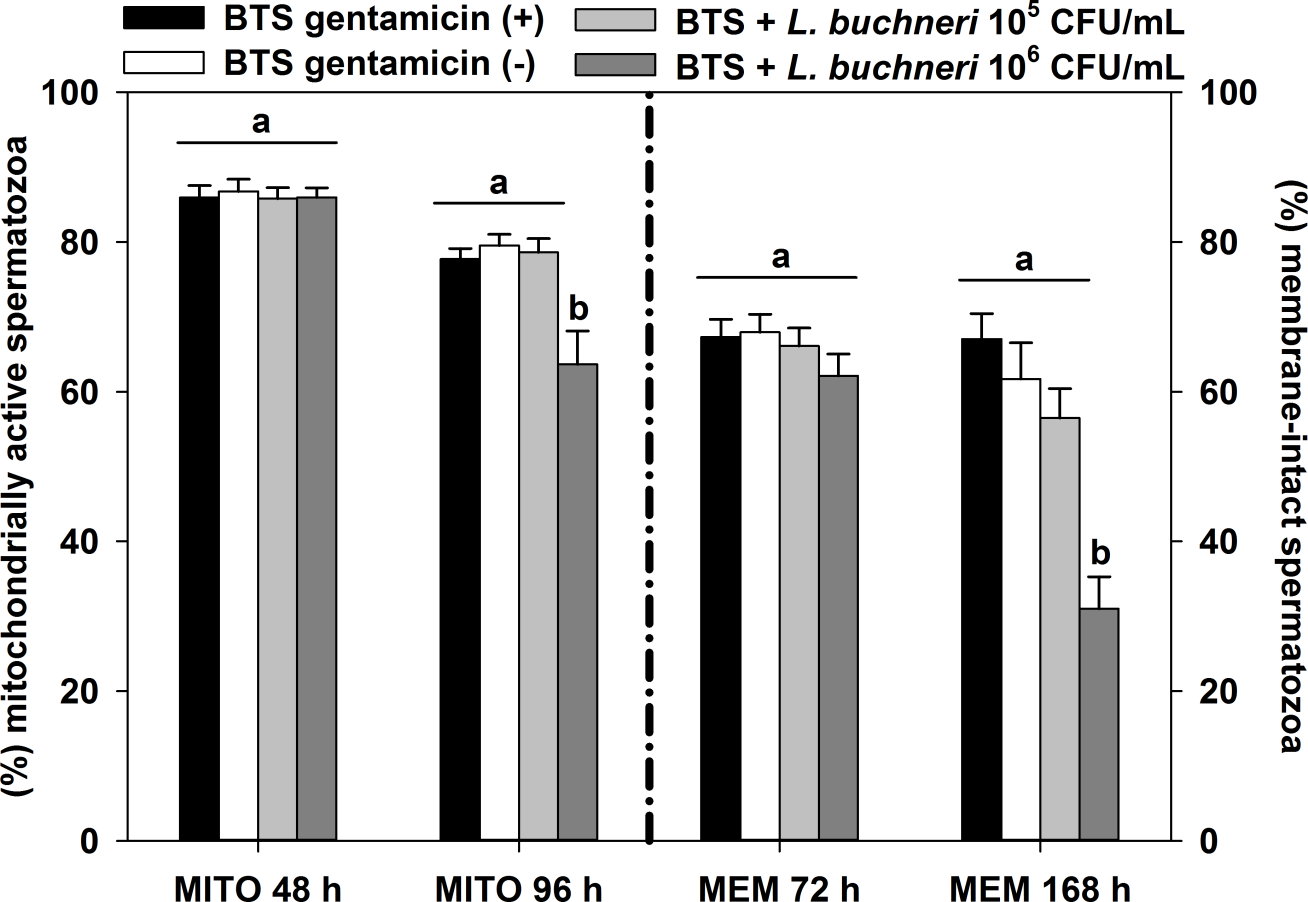
Effect of 10^5^ and 10^6^ CFU/mL *L*. *buchneri* DSM 32407 during long-term incubation in BTS-extended boar semen on mitochondrially active (MITO: 48 and 96 hours) and membrane-intact (MEM: 72 and 168 hours) spermatozoa. Normospermic ejaculates of 12 fertile boars were extended to 2.4 × 10^7^ spermatozoa/mL in BTS with 250 μg/mL gentamicin (positive control (+)) and without gentamicin (negative control (-)). Extended semen was stored in 100 mL semen bottles (Minitüb) for seven days at 17°C. Results are represented as mean ± SEM (n = 12 per group). Bars with different letters indicate significant differences between groups (*P* < 0.05).

The percentage of mitochondrially active spermatozoa after 96 h (*P* = 0.009) and membrane-intact spermatozoa after 168 h (*P* < 0.001) of semen incubation in the presence of LAB was significantly lower with 10^6^ CFU/mL *L*. *buchneri* DSM 32407 when compared with all other groups (Fig 3). Furthermore, the presence of 10^5^ CFU/mL *L*. *buchneri* DSM 32407 did not affect percentage of mitochondrially active spermatozoa and membrane-intact spermatozoa compared with the control groups (BTS with gentamicin and BTS w/o gentamicin).

**Fig 4:**
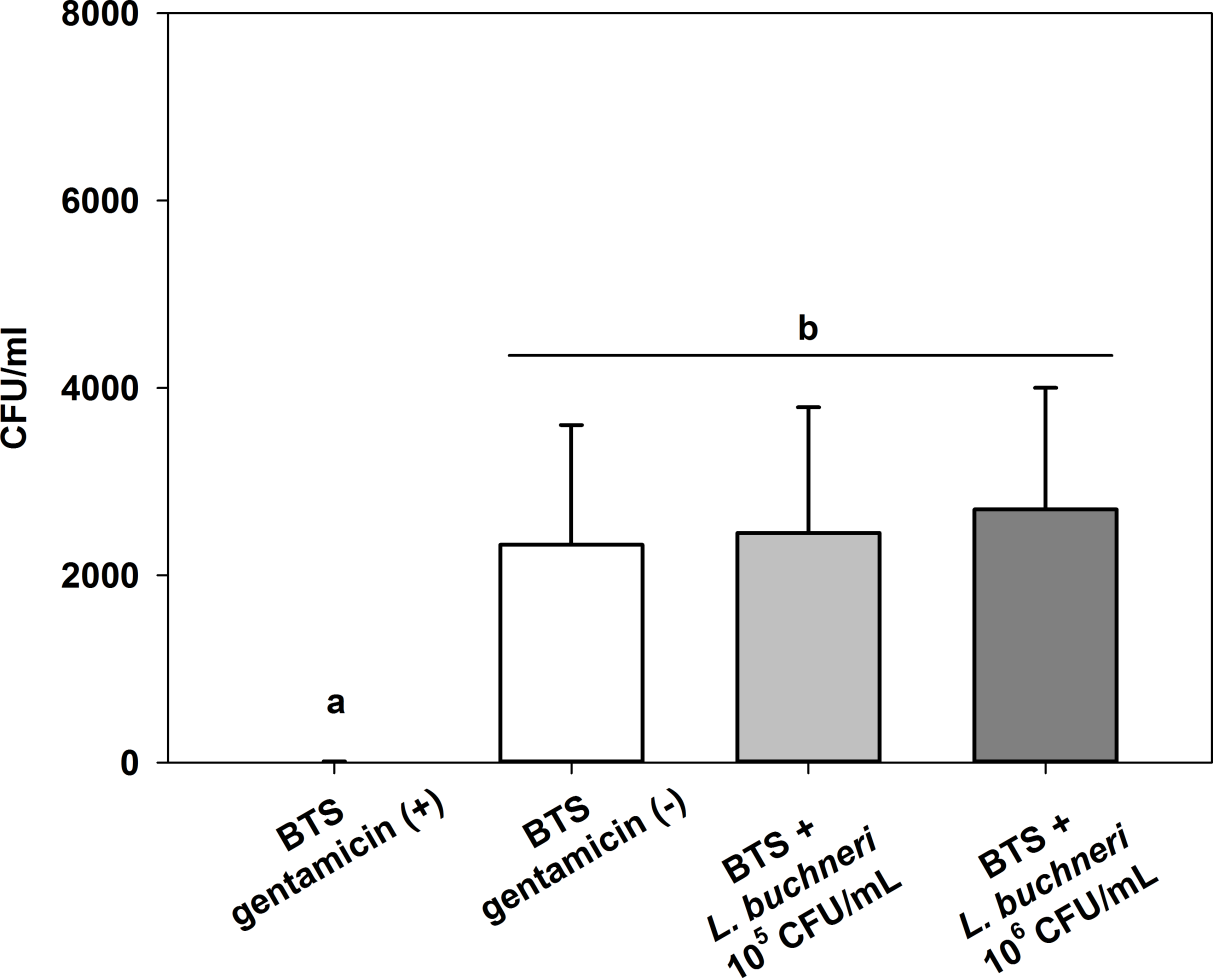
Effect of 10^5^ and 10^6^ CFU/mL *L*. *buchneri* DSM 32407 on the total amount of bacteria (without lactobacilli) after 48 h of semen storage in BTS-extended boar semen. Normospermic ejaculates of 12 fertile boars were extended to 2.4 × 10^7^ spermatozoa/mL in BTS with 250 μg/mL gentamicin (positive control (+)) and without gentamicin (negative control (-)). Extended semen was stored in 100 mL semen bottles (Minitüb) at 17°C. Results are represented as mean ± SEM (n = 12 per group). Bars with different letters indicate significant differences between groups (P < 0.05).

The addition of *L*. *buchneri* DSM 32407 to BTS-extended boar semen had no competitive effect on the total amount of bacteria 48 h after semen preservation (Fig 4). BTS with gentamicin was able to prevent bacterial growth and therefore differed significantly (*P* = 0.032) from the groups without gentamicin independently of the addition of *L*. *buchneri* DSM 32407.

Monitoring of pH revealed a significant difference (*P* < 0.001) between samples stored at 17°C without warming for up to 72 h and *L*. *buchneri* DSM 32407-supplemented samples that were incubated at 38°C for 300 min after storage of 168 h at 17°C. During storage, pH values did not differ significantly between control and 10^5^ or 10^6^ *L*. *buchneri* DSM 32407 CFU/ml. Warming of the samples resulted in acidification of *L*. *buchneri* DSM 32407-supplemented groups. This acidification was even more pronounced for 10^6^ than for 10^5^ CFU/mL (pH values of 5.79 ± 0.21 and 6.29 ± 0.58, respectively), but both groups differed significantly (*P* < 0.001) from the control (7.48 ± 0.04).

## Discussion

Several different LAB strains were isolated from 111 fresh cryopreserved boar ejaculates. The composition and number of LAB in boar ejaculates is most likely diverse, analogous to the vaginal flora in sows. In most animals, the vaginal milieu is characterized by an acidic pH value and a bacterial flora mainly consistent with LAB species [25]. The concentration of LAB in vaginal secretion varies between 10^5^ and 10^8^ CFU/mL. This is in the same range as observed in seminal plasma of about 10^6^ CFU/mL [42]. LAB are described as part of a biosystem of apathogenic and pathogenic bacteria in equilibrium [43]. The contribution of LAB to maintain or restore a healthy vaginal flora in humans and animals has been the aim of an increasing number of research projects and is still not completely elucidated [32,33,44].

Three of the isolated bacterial strains from fresh cryopreserved boar semen (*L*. *animalis*, *L*. *reuteri* and *Weisella minor*) could be cultivated in liquid culture in a sufficient manner. Most likely, an isolation from fresh ejaculates could result in more and different strains. It is known that some LAB are very sensitive to environmental conditions and are only able to grow in sufficient amounts on an agar plate but not in liquid culture under the chosen standard conditions. However, in one study using fresh semen samples, LAB could be obtained in each sample but with only three samples being investigated, the significance of this study is very limited [42]. In another study using fresh bovine endometrial samples, almost every strain that separated on agar plates was able to grow in liquid culture in a sufficient manner [27]. As a control, the heterologous strain *L*. *buchneri* DSM 32407, which was isolated from the bovine uterus was chosen [27]. This distinct strain was included in this study because it grew very easily in a liquid culture system and neither affected the viability nor provoked an inflammatory response of the bovine endometrial cells *in vitro* [27]. Furthermore, this specific strain also improved the reproductive performance in cows with signs of subclinical endometritis [45].

In pig production, artificial insemination is widely carried out and the use of fresh diluted semen is predominant. For this reason, there are increasing interests in developing new extenders and in establishing the optimal storage conditions for diluted spermatozoa [46,47]. For short- and long-term co-cultivation of spermatozoa with LAB, a commercial BTS extender was chosen because it warrants preservation during storage for three days [5]. In this extender, two of the used LAB (*L*. *animalis* and *L*. *buchneri* DSM 32407) were able to grow. However, the best results for growth were observed with the lactose-containing extender Androstar CryoPlus, but this extender is normally only used for cryopreservation. The short-term co-cultivation of spermatozoa with *L*. *animalis* led to significant negative effects regarding the proportion of progressively motile spermatozoa but neither mitochondrial activity nor membrane status were affected negatively. In addition, *L*. *animalis* and *L*. *buchneri* DSM 3240 caused a slight but significant pH shift of the preservation medium. Therefore, such findings of decreased sperm quality could be explained for *L*. *animalis* with the metabolic products of this strain because *L*. *buchneri* DSM 32407 did not affect the sperm quality under these conditions. These observations are similar to recent findings with the strains *L*. *brevis*, *L*. *salivarius* and *L*. *plantarum*, whose presence did not affect human sperm viability and motility after 30 min incubation time [48].

Based on the results of the first experiments, *L*. *buchneri* DSM 32407 was chosen as the most promising strain for long-time co-cultivation of 7 days in a concentration of 10^5^ and 10^6^ CFU/mL. After three days of storage, negative impacts on sperm quality began to occur in the higher concentration of 10^6^ CFU/mL. Overall, motility after 96 h, thermo-resistance after 72 h and 168 h, mitochondrial activity after 96 h and membrane integrity after 168 h of storage were affected. Especially during long-term co-incubation, the TRT showed negative concentration dependent effects of *L*. *buchneri* DSM 32407. This implies a potentially negative impact on the fertility of boar semen supplemented with high concentrations of LAB.

However, no difference in semen quality could be detected with 10^5^ CFU/mL *L*. *buchneri* DSM 32407 compared with the BTS w/o gentamicin. Contrary to our expectations, we could not determine a quantitative displacement of contaminant bacteria by inoculation with *L*. *buchneri* DSM 32407 during long-time semen co-incubation. During 300 min of incubation at 38°C, there was a marked acidification of LAB-supplemented samples. Very likely, this is a result of the bacteria’s metabolic activity, reducing sugars to lactic acid. This acidification might be a reason for the lower sperm quality in LAB-supplemented samples. As *L*. *buchneri* DSM 32407 is known to be hetero-fermentative, we also checked potential alcohol content (data not published). As no detectable amount of alcohol was produced in any group, this explanation for deterioration of sperm quality may be rejected.

In a recent study, the human endometrial microbiota was investigated. It showed that women with a *Lactobacillus*-dominated-microbiota (> 90%) have higher chances of implantation, pregnancy and live birth after *in vitro* fertilization [49]. In this context, a positive effect of intrauterine lactobacilli on fertility might also be related to stimulatory effects on the blastocyst around the time of implantation. *In vitro* experiments showed that *L*. *acidophilus* culture supernatant positively influenced the growth and development of bovine embryos [50].

Such an influence on bovine genital health was additionally observed in recent studies. The weekly administration of a mixture of lactobacilli into the vagina from two weeks before until four weeks after parturition (six treatments) decreased the occurrence of purulent vaginal discharge in dairy cows at week three postpartum [51]. Furthermore, *L*. *buchneri* DSM 32407 resulted in shorter days open in cows with signs of subclinical endometritis after intra-uterine application [45].

In general, LAB metabolize sugars, producing lactic acid and carbon dioxide (CO_2_) and lowering the pH value of the medium. The acid medium is a strategy of LAB to inhibit growth of competing bacteria in the same medium. Additional to lactic acid and CO_2_, they produce ethanol (from hexoses) and acetic acid (from pentoses). Furthermore, some LAB generate antimicrobial peptides, which are especially effective against Gram-positive bacteria. Unfortunately, these peptides are inactive in media with basic pH values [52]. Another essential characteristic of some LAB is the production of hydrogen peroxide (H_2_O_2_). H_2_O_2_ is antiseptic due to its oxidative effect and stabilizes, complementary to lactic acid, the physiologic Lactobacillus flora, protecting these bacteria from the influence of others [52]. All of these factors, alone or in combination with each other, may be the reason for a spermicidal effect of LAB. In future research, LAB metabolism and their competition with pathogens for limited nutrients have to be taken into account [53]. The question, which role physiologically incorporated LAB play in fresh boar ejaculates remains unclear. Among topics for future research, it will be important to determine whether homeostatic mechanisms involve direct interactions between spermatozoa and LAB.

## Conclusions

The results of the present study demonstrate that there are *Lactobacillus* species present in the porcine seminal plasma, which can be cultivated using standard procedures. Most notably, *L*. *buchneri* DSM 32407 did not affect the sperm motility and viability in such an approach. However, long-term co-incubation with LAB had a negative influence on spermatozoa, particularly after warming. Such *Lactobacillus* species however, may not affect the motility and viability of spermatozoa *in vivo*, which the results from the short term incubation with extended boar semen suggest.
